# Molecular identification and characterization of *Anaplasma capra* and *Anaplasma platys*-like in *Rhipicephalus microplus* in Ankang, Northwest China

**DOI:** 10.1186/s12879-019-4075-3

**Published:** 2019-05-17

**Authors:** Wen-Ping Guo, Bing Zhang, Yi-Han Wang, Gang Xu, Xiaoquan Wang, Xuebing Ni, En-Min Zhou

**Affiliations:** 1 Department of Preventive Veterinary Medicine, College of Veterinary Medicine, Northwest A&F University, Xinong Road 22, Yangling, 712100 Shaanxi China; 2 Scientific Observing and Experimental Station of Veterinary Pharmacology and Diagnostic Technology, Ministry of Agriculture, Yangling, Shaanxi China; 3 Department of Human parasitology, Preclinical Medicine College, Xinjiang Medical University, Urumqi, Xinjiang China; 4 Xuwang Town Comprehensive Agricultural Service Station, Hanzhong, Shaanxi China; 5 State Key laboratory of Emerging infectious disease, School of Public Health, The University of Hong Kong, Hong Kong, China

**Keywords:** *Anaplasma capra*, *Anaplasma platys*-like strains, China, *Rhipicephalus microplus* ticks, *Rrs*, *gltA*, And *groEL* genes

## Abstract

**Background:**

Four species within *Anaplasma* genus are emerging zoonotic pathogens, which are transmitted by ticks and generate veterinary and public health concerns. Here, we performed a molecular survey of *Anaplasma* in Ankang, Northwest China.

**Methods:**

Hard ticks were collected and identified using morphological and molecular methods. Human-pathogenic *Anaplasma* species were tested using nested polymerase chain reaction. The nearly complete *rrs*, *gltA*, and *groEL* genes sequences from revealed *Anaplasma* species were amplified and sequenced to determine their molecular characteristics and their phylogeny.

**Results:**

All ticks collected in Ankang belonged to the *Rhipicephalus microplus*. Novel unclassified *Anaplasma* strains genetically related to *A. platys* and *A. capra* were detected in these ticks. Co-infection of these two organisms was also found. The novel unclassified *Anaplasma* strains identified in this study formed a distinct phylogenetic lineage based on the *groEL* gene and two lineages based on the *gltA* gene within *A. platys* and related strains group. The revealed *A. capra* strains identified in this study were most closely related to those detected in humans and other vertebrate animals.

**Conclusion:**

We revealed the presence of *A. capra*, a novel human pathogens in *R. microplus* ticks in previously unrecognized endemic regions. We also detected a novel unclassified *Anaplasma* species genetically related to *A. platys*. The epidemiology of anaplasmosis caused by these two *Anaplasma* species in humans should be assessed in future studies.

**Electronic supplementary material:**

The online version of this article (10.1186/s12879-019-4075-3) contains supplementary material, which is available to authorized users.

## Background

*Anaplasma* genus comprises a group of Gram-negative obligate intracellular bacteria that inhabit diverse eukaryotic hosts. Seven classified species have been described in this genus so far [1–3]. In the last two decades, the rapidly spreading diseases caused by *Anaplasma* in humans and livestock have received increasing attention since the first incidence of human granulocytic anaplasmosis (HGA) caused by *A. phagocytophilum* was reported [4]. During this period, many known and emerging *Anaplasma* species have been proved to be pathogenic to humans, causing anaplasmosis or associated diseases. For instance, *A. ovis* and *A. platys* have been identified as human pathogens [5, 6], and *A. capra*, a novel *Anaplasma* species, was also confirmed to be a human pathogen in China [3]. Among these causative agents, *A. phagocytophilum* is the most common pathogen with a worldwide distribution [1, 7], while others are usually neglected because the cases occur only sporadically.

Since 1982, several classified species and unclassified strains belonging to *Anaplasma* genus have been reported in ticks and mammals from China [3, 8, 9]. Human-pathogenic species and unclassified strains within *Anaplasma* have been identified, including *A. capra*, *A. ovis*, *A. phagocytophlum*, and *A. platys*. To date, only HGA caused by *A. phagocytophilum* has been reported [10]. In contrast, less attention has been paid to the epidemiology and genetic characteristics of other pathogens, although they have been reported in ticks and livestock. In Northwest China, molecular epidemiological surveys confirmed the presence of the four *Anaplasma* species mentioned above: *A. phagocytophilum* and *A. ovis* in Qinghai Province; *A. ovis* in Tibet [11]; *A. platys*, *A. ovis*, and *A. capra* in Gansu Province [12–14]; and *A. platys* and *A. ovis* in Xinjiang Uygur Autonomous Region [15–17].

Shaanxi Province is located in Northwest China, forming a gateway to four other provinces in this region and connecting the East, Central, and Northwest China. Previous studies have revealed the presence of at least 26 tick species in this region [18], but none of them has attempted to molecularly characterize the human-pathogenic *Anaplasma* species that they may host. To know more about the circulation and the genetic characteristic of human-pathogenic *Anaplasma* species in Shaanxi, we sampled ticks from domestic animals in Ankang city and screened them for *Anaplasma* spp.

## Methods

### Collection of tick samples

**Fig. 1:**
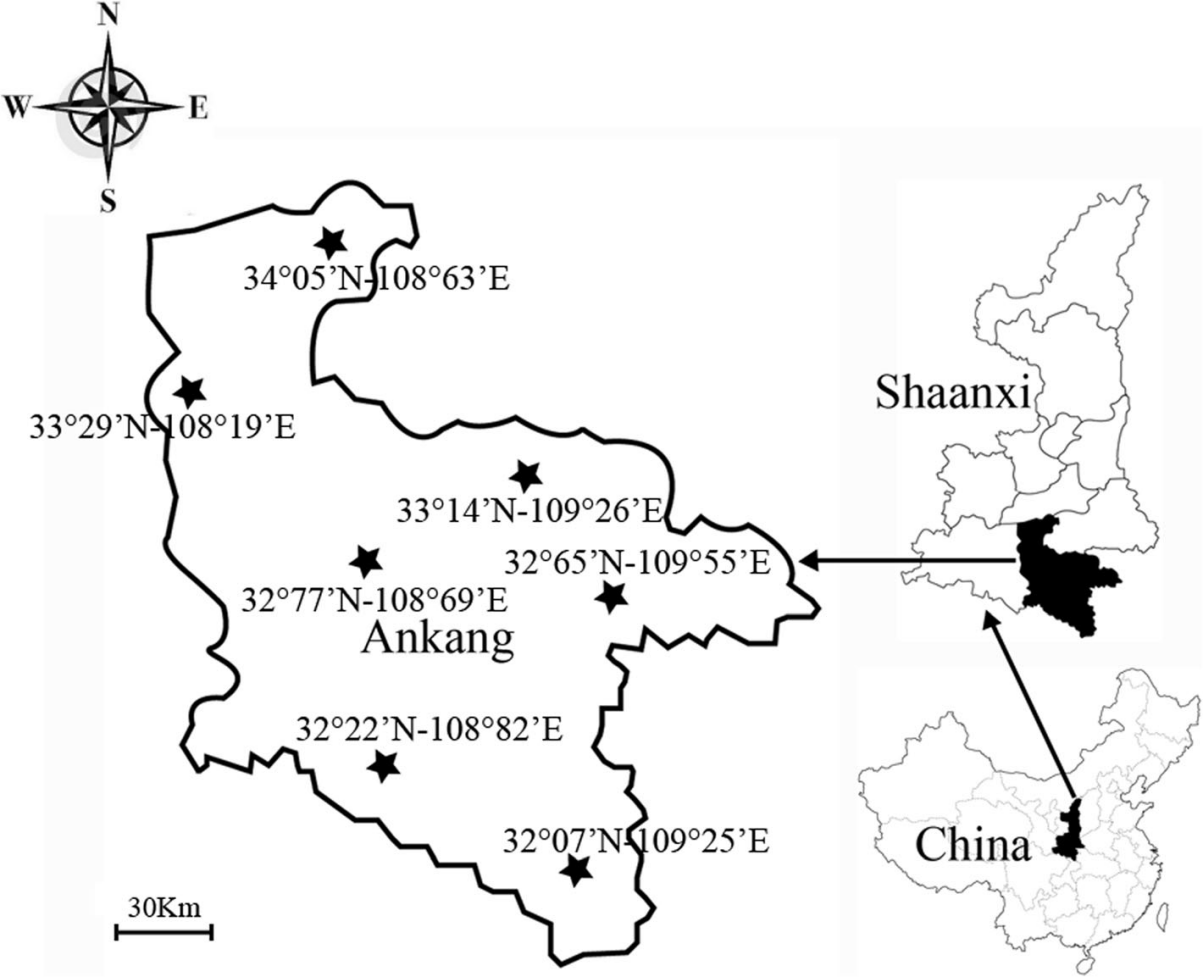
Map with the coordinates of all seven sampling sites (★) in Ankang, Shaanxi Province, China. The maps of Ankang city and Shaanxi Province was generated using ArcGIS (version 10.0; http://www.arcgis.com/index.html) and edited using Adobe Illustrator. The map of China was obtained from http://english.freemap.jp/item/asia/china.html

Ankang city is located in Northwest China, southeast of Shaanxi Province and south of the Qinling Mountains (31°42′ to 33°50′N, 108°00′ to 110°12′E). Its topography is characterized by densely vegetated mountains and hills, and livestock often become heavily infested with ticks when they graze in the field. In this study, ticks were sampled in 2017 and 2018 from the body surface of cattle, goats, or sheep, and one tick per animal was collected from seven sampling sites (Fig. 1). These animal hosts of ticks were privatedly-owned and verbal consent was obtained from the owners of these animals for tick samples to be taken. The ticks were first identified by morphology [19, 20], and the identification of one-quarter of adults and all nymphs was further confirmed by analyzing 710-bp of the *COI* gene using the LCO1490 and HCO2198 primers [21]. The Institutional Animal Care and Use Committee of Northwest A&F University have reviewed and approved this study.

### DNA extraction and detection of *Anaplasma* species

The total DNA was extracted from the whole body of each tick. The ticks were first washed with 75% alcohol and then with phosphate-buffered saline. After washing, the total DNA was extracted from individual ticks using Tissue DNA Kit (Omega, Norcross, GA, USA) according to the manufacturer’s instructions. The DNA samples were diluted in 80 μL of the elution buffer and stored at − 20 °C.

The extracted DNA samples were initially screened for the presence of *Anaplasma* spp. by amplifying the *rrs* gene using nested polymerase chain reaction (PCR) with primers specific for *A. phagocytophilum*, *A. ovis*, and *A. capra* as described previously [22–26]. The primers PLATYS [27], Ehr2, and Ehr4 [28] were used to detect *A. platys* by semi-nested PCR, also targeting the *rrs* gene. The primer sequences are shown in the Table 1. Double-distilled water was used as a negative control, and the extracted DNA samples positive for these four *Anaplasma* species were used as positive controls. To avoid contamination, the DNA extraction, PCR mixture preparation, template addition, and agarose gel electrophoresis were performed in a fume hood in four separate rooms and filter tips were used in these processes.

**Table 1:** Primer sequences used in this study for detection of *Anaplasma* species pathogenic to humans

Pathogens	Target gene	Primer	Oligonucleotide sequences (5′- 3′)	References
*A. phagocytophilum*	*rrs*	EE1	TCCTGGCTCAGAACGAACGCTGGCG (+)	Barlough et al. [22]
		EE2	AGTCACTGACCCAACCTTAAATGGCTG (−)	Barlough et al. [22]
		SSAP2f	GCTGAATGTGGGGATAATTTAT (+)	Kawahara et al. [23]
		SSAP2r	ATGGCTGCTTCCTTTCGGTTA (+)	Kawahara et al. [23]
*A. capra*	*rrs*		GCAAGTCGAACGGACCAAATCTGT (+)	Yang et al. [26]
			CCACGATTACTAGCGATTCCGACTTC (−)	Yang et al. [26]
		Ehr3	TGCATAGGAATCTACCTAGTAG (+)	Rar et al. [28]
		Ehr4	CTAGGAATTCCGCTATCCTCT (−)	Rar et al. [28]
*A. ovis*	*rrs*	EE1	TCCTGGCTCAGAACGAACGCTGGCG (+)	Barlough et al. [22]
		EE2	AGTCACTGACCCAACCTTAAATGGCTG (−)	Barlough et al. [22]
		297E	ACACGGTCCAGACTCCTACG (+)	Ochirkhuu et al. [24]
		1144R	CTTGACATCATCCCCACCTT (−)	Ochirkhuu et al. [24]
*A. platys*	*rrs*	PLATYS	GATTTTTGTCGTAGCTTGCTATG (+)	Martin et al. [27]
		Ehr2	AGTAYCGRACCAGATAGCCGC (−)	Rar et al. [28]
		Ehr4	CTAGGAATTCCGCTATCCTCT (−)	Rar et al. [28]

### Amplification of nearly complete *rrs*, and partial/nearly complete *groEL* and *gltA* genes

The positive samples were used to amplify the nearly complete *rrs* gene, and the molecular characteristics of the revealed *Anaplasma* strains were analyzed using partial and/or nearly complete *groEL* and *gltA* genes. The nearly complete *rrs*, *gltA*, and *groEL* genes were obtained by amplifying two overlapping fragments by semi-nested PCR (Additional file 1: Figure S1).

For *A. capra*, the former fragment was amplified with fD1/Ehr2 primer pairs for the primary round and fD1/Ehr4 for the secondary round of semi-nested PCR, and the latter fragment was amplified with Ehr1/rp2 for the primary round and Ehr3/rp2 for the secondary round of semi-nested PCR [25, 28]. The same strategy was used to amplify the almost full-length *rrs* gene of *A. platys* and related strains, and only the primer PLATYS primer was used instead of fD1.

The partial *gltA* gene of *A. platys* and related strains was amplified with Pglt-F/Pglt-R1 primers for the primary round and Pglt-F/Pglt-R2 for the secondary round of semi-nested PCR. The partial *groEL* gene were amplified with Pgro-F1/Pgro-R primers for the primary round and Pgro-F2/Pgro-R for the secondary round of semi-nested PCR (Additional file 1: Figure S1).

The nearly complete *groEL* and *gltA* genes of *A. capra* were obtained according to Guo et al. [29]. By amplifying two overlapping fragments, almost-full-length *groEL* and *gltA* genes of *A. platys* and related strains were amplified using semi-nested PCR (Additional file 1: Figure S1). The former fragment of *gltA* was amplified with Pglt-F/Pglt-R1 primer pairs for the primary round and Pglt-F/Pglt-R2 for the secondary round of semi-nested PCR, whereas the latter fragment of *gltA* was amplified with Pglt-L-F1/Pglt-L-R for the primary round and Pglt-L-F2/Pglt-L-R for the secondary round*.* For the nearly complete *groEL* gene, the former fragment was amplified with Pgro-F-F/Pgro-F-R1 for the primary round and Pgro-F-F/Pgro-F-R2 for the secondary round of semi-nested PCR, and the latter fragment was amplified with Pgro-L-F1/Pgro-L-R for the primary round and Pgro-L-F2/Pgro-L-R for the secondary round of semi-nested PCR. All novel primer sequences in this study (Table 2) were designed based on the known sequences of each *Anaplasma* species.

**Table 2:** Primer sequences designed to amplify the *gltA* and *groEL* genes in this study

Pathogens	Target gene	Oligonucleotide sequences (5′- 3′)	Fragment
*A. platys*	*gltA* (partial)	Pglt-F: ATGAWAGAAAAWGCTGTTTT (+)	/
		Pglt-R1: TCATGRTCTGCATGCATKATG (−)	
		Pglt-R2: CATGCATKATGAARATMGCAT (−)	
	*groEL* (partial)	Pgro-F1: TTGATCATCGCTGAAGACGT (+)	/
		Pgro-F2: ACTCTCGTCTTGAACAAGCT (+)	
		Pgro-R: CCACTCTGTCTTTACGCTCT (−)	
	*gltA* (nearly complete)	Pglt-F: ATGAWAGAAAAWGCTGTTTT (+)	Former
		Pglt-R1: TCATGRTCTGCATGCATKATG (−)	
		Pglt-R2: CATGCATKATGAARATMGCAT (−)	
		Pglt-L-F1: GATGCWCATCCYATSGCMATGT (+)	Latter
		Pglt-L-F2: CGTGMTSGCTATAGCGMAART (+)	
		Pglt-L-R: TCAYACCATTGDGAYRCCCAT (−)	
	*groEL* (nearly complete)	Pgro-F-F: AAATGKCAAATACGGTWGTC (+)	Former
		Pgro-F-R1: ACAACACCTTCCTCKACAGC (−)	
		Pgro-F-R2: CTGKCTTTRCGYTCTTTAACTTC (−)	
		Pgro-L-F1: GAYGGTATGCAGTTTGATCGCG (+)	Latter
		Pgro-L-F2: ATGCAGTTTGATCGCGGWTATC (+)	
		Pgro-L-R: CAGCRAGGTCGAAYGCAATAC (−)	

### Cloning and sequencing of the PCR products

The amplified DNA was electrophoresed in 1.0% agarose gels. All the PCR amplicons of expected size were purified using Gel Extraction kit (TaKaRa, Dalian, China). Purified PCR products were ligated with the cloning vector pMD19-T (TaKaRa, Dalian, China), and the recombinant vector was transformed into competent *E. coli* cells. Finally, positive clones were identified by PCR and at least three positive clones were sequenced for each PCR product to determine the consensus sequence.

### Analysis of obtained sequences

Two overlapping fragments were edited and assembled using Bioedit v.7.1.11 to obtain the nearly complete gene sequences [30]. Multiple alignments of DNA sequences of the recovered *rrs*, *gltA,* and *groEL* genes were performed using the ClustalW method in the MEGA 7.0 software with reference sequences from GenBank [31]. The nucleotide identities were calculated using the MegAlign program in Lasergene [32]. The best-fit nucleotide substitution model was determined using MEGA 7.0 to reconstruct the phylogenetic trees based on *rrs*, *gltA* and *groEL* [31]. Phylogenetic trees based on the maximum likelihood method were reconstructed by PhyML v3.2 [33], with 100 replicates for bootstrap analysis. The trees were midpoint-rooted and visualized by MEGA 7.0, presenting a bootstrap value of more than 70%. The sequences that were newly generated for phylogenetic analysis in this study were submitted to GenBank and assigned accession numbers MH716407–MH716436 and MH762071–MH762085.

## Results

### Collection of samples and identification of tick species

In the summer and autumn of 2017 and 2018, 397 adult and 59 nymph ticks were collected from livestock (65 sheep, 113 goats, and 278 cattle) in rural villages of Ankang, China. Based on the morphology, all collected ticks were identified as *Rhipicephalus microplus*, and the identification was confirmed molecularly by amplifying the *COI* gene isolated from 59 nymph and 100 adult ticks. After sequencing of the PCR products, these sequences shared the highest identities (99.3–99.6%) with those of *R. microplus*, confirming that all collected ticks belonged to this species. Seven representative *COI* gene sequences selected from each group sharing 100% identities were submitted to GenBank as MK371424–MK371430.

### Identification and genetic analyses of *Anaplasma* spp. in ticks

In the screening of *Anaplasma* spp. using partial *rrs* gene, 167 PCR products of expected size were obtained using primers specific to *A. capra* and 35 using those specific to *A. platys*. All these PCR products were sequenced. The 167 partial *rrs* gene sequences shared 99.3–99.7% nucleotide identities with those of *A. capra*, and the other 35 shared 99.1–99.6% nucleotide identities with those of *A. platys* and/or related strains complex. Co-infection with these two bacteria was found in 20 ticks. However, *A. phagocytophilum* and *A. ovis* were not detected in the analyzed ticks.

### Phylogenetic relationship of the revealed *A. platys*-like bacteria with known strains

**Fig. 2:**
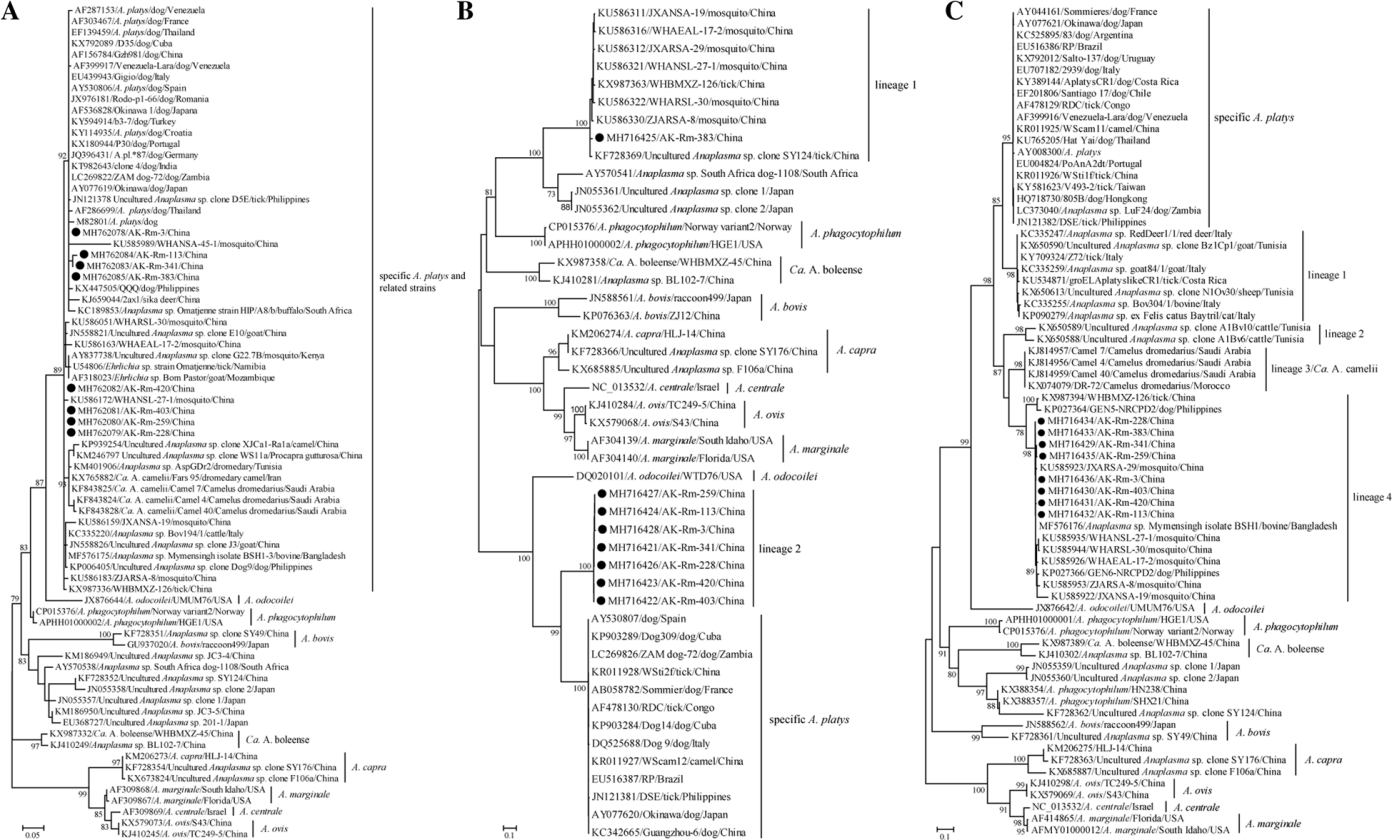
Phylogenetic position of revealed *A. platys-*like strains within *Anaplasma* genus. **a** Phylogenetic trees based on the *rrs* gene; (**b**) phylogenetic trees based on the *gltA* gene; (**c**) phylogenetic trees based on the *groEL* gene. Numbers at each node indicate bootstrap values. All three trees were rooted by mid-point methods. The scale bar represents the number of nucleotide substitutions per site. The taxa marked by circles depict the sequences obtained in this study

To better understand the phylogenetic relationships between the *A. platys* variants in this study and those described previously, the *rrs* gene sequences with the length of 1300 bp were amplified successfully from three representative positive samples. These three *rrs* gene sequences shared 100% nucleotide identities with each other, and 99.1–99.9% with known *rrs* gene sequences of specific *A. platys, Candidatus* A. camelii [34], and unclassified *Anaplasma* strains genetically related to *A. platys* (Additional file 2: Table S1). In the *rrs* tree, all these sequences (found in ticks, mosquitoes, dogs, goats, and cattle) had a close relationship with each other and formed a big cluster (named “specific *A. platys* and related strains”) separated from other species (Fig. 2a).

In addition to the *rrs* gene sequences, partial *gltA* gene sequences (about 600 bp) were recovered from all positive samples, and two nearly complete sequences (about 1150 bp) were recovered from the representative positive samples. For the partial *gltA* gene, it is notable that 34 strains (except AK-Rm383) shared 99.3–100% nucleotide identities and were distantly related to other known *A. platys* and related sequences, showing only 57.3–77.8% nucleotide identities (Additional file 2: Table S1). However, AK-Rm383 presented 96.3–97.9% nucleotide identities with variants identified in mosquitoes from Wuhan [35], and WHBMXZ-126 was also detected in *R. microplus* from Wuhan [36]. An uncultured *Anaplasma* sp. clone SY124 from *Haemaphysalis longicornis* tick in Shenyang [37] presented 52.6–60.3% nucleotide identities with other variants, including 34 strains recovered herein (Additional file 2: Table S1).

Consistent with the similarity analysis, all the specific *A. platys* and related strains were divided into three lineages in the phylogenetic tree of the *gltA* gene (Fig. 2b). All the specific *A. platys* strains formed a distinct lineage. The first lineage consisted of AK-Rm383, WHBMXZ-126, uncultured *Anaplasma* sp. clone SY124, and several sequences identified in mosquitoes from Wuhan. The second lineage included seven representatives from 34 strains obtained in the present study. In the *gltA* tree, the first distinct lineage was distant to *Anaplasma* sp. South Africa dog-1108 [38], and the uncultured *Anaplasma* sp. clone 1 and clone 2 [39]. The specific *A. platys* lineage was distant to the second one although they clustered together, sharing 74.0–77.2% identities. The position of *Ca*. A. camelii in the *gltA*-based tree was not determined due to the unavailability of its *gltA* gene.

In addition, partial *groEL* sequences (380 bp) were obtained from all positive samples, and five nearly complete sequences (about 1450 bp) were sequenced from selected positive samples. For the *groEL* gene, the partial sequences showed 99.7–100% nucleotide identities, and the nearly complete sequences presented 99.5–99.7% identities. In the *groEL* gene tree, all specific *A. platys* and related strains were divided into five lineages (Fig. 2c), which all clustered together. Furthermore, all eight representatives from 34 strains obtained in the present study (including AK-Rm383, WHBMXZ-126, *Anaplasma* sp. Mymensingh isolate BSH1 [40], several strains identified in mosquitoes from Wuhan, and two strains identified in dogs from the Philippines), clustered and formed the fourth lineage, whereas uncultured *Anaplasma* sp. clone SY124 clustered with HN238 and SHX21 rather than with the specific *A. platys* and related strains. All specific *A. platys* strains formed a distinct lineage, similar to that in the *gltA* tree. The first lineage composed by *A. platys*-like strains infecting Italian and Tunisian ruminants [41, 42] and Italian cats [43], as well as of *A. platys*-like strains detected in ticks from Italy [44] and Costa Rica [45]. The second lineage composed by uncultured *Anaplasma* sp. clones A1Bv10 and A1Bv6 detected in cattle from Tunisia, which were considered as *A. platys*-like strains by Ben Said et al. [41]. It is notable that *Ca*. A. camelii formed the third lineage and also fell into the diversity of the specific *A. platys* and related strains in the *groEL* tree, similar to that in the *rrs* tree. The nucleotide identities among specific *A. platys* and another four lineages of *A. platys-*like strains are shown in Table 3 and Table S1.

**Table 3:** The nucleotide identities of *groEL* gene among specific *A. platys* and related four lineages

	Specific *A. platys*	lineage 1	lineage 2	lineage 3 (Ca. A. camelii)	lineage 4
Specific *A. platys*		86.4–88.6%	92.4–93.2%	91.5–92.8%	84.8–86.7%
lineage 1			84.8–86.3%	85.0–88.9%	87.3–90.9%
lineage 2				–	86.3–88.0%
lineage 3 (Ca. A. camelii)					–
lineage 4					

### Phylogenetic relationship of *A. capra* in this study with known strains

**Fig. 3:**
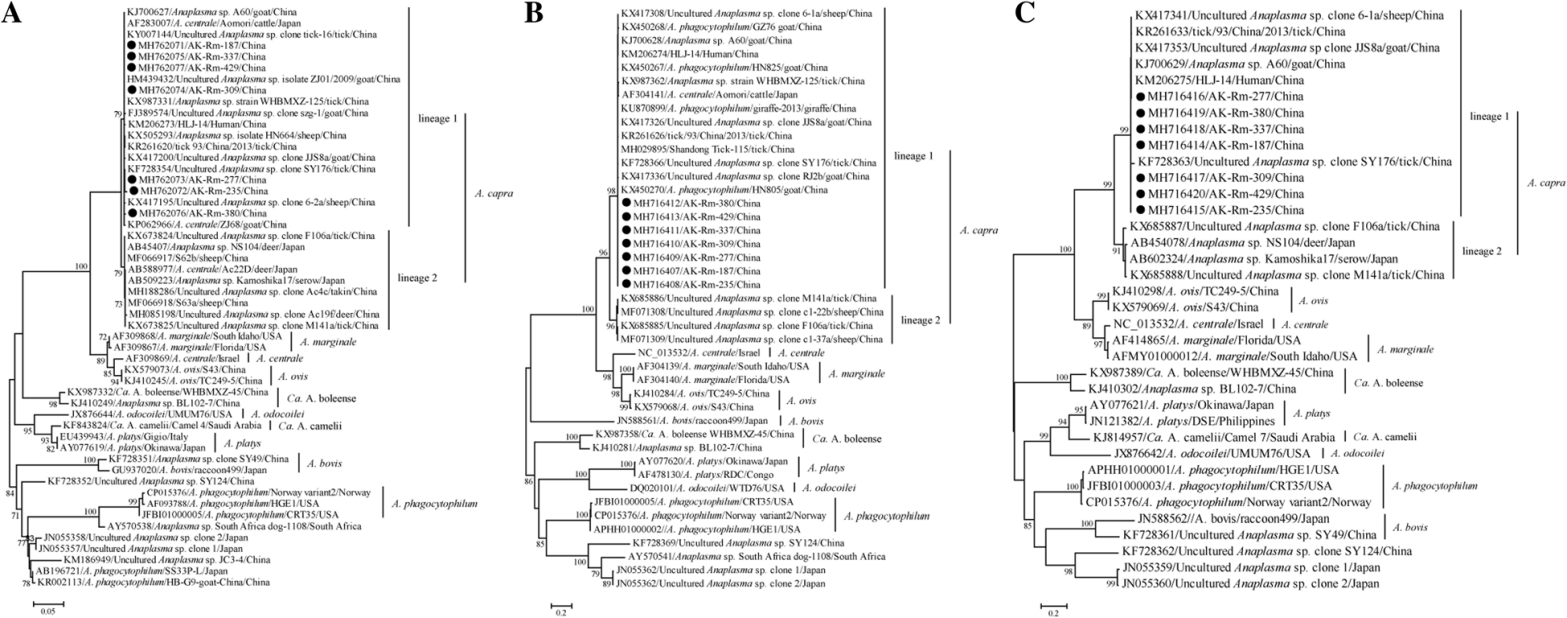
Phylogenetic position of *A. capra* within *Anaplasma* genus. **a** Phylogenetic trees based on the *rrs* gene; (**b**) phylogenetic trees based on the *gltA* gene; (**c**) phylogenetic trees based on the *groEL* gene. Numbers at each node indicate bootstrap values. All three trees were rooted by mid-point methods. The scale bar represents the number of nucleotide substitutions per site. The taxa marked by circles depict the sequences obtained in this study

Almost full-length *rrs* (about 1400 bp), and partial *gltA* (about 1100 bp) and *groEL* (about 1450 bp) DNA sequences were obtained from seven *A. capra* positive samples selected randomly. A comparison of the *rrs* gene sequences showed that all sequences had 99.6–100% nucleotide identity with each other and 99.0–100% nucleotide identities with known *A. capra* sequences (Additional file 2: Table S1). In the *rrs* tree, all revealed sequences clustered in the first lineage together with those of *A. capra* strains detected in goats, sheep, ticks, deer, and humans from China [3] and cattle (previously defined as *A. centrale*) from Japan (Fig. 3a). The second lineage included *A. capra* sequences detected in ticks, sheep, deer from China, and deer and serows from Japan.

The topology of the partial *groEL*- and *gltA*-based phylogenetic trees resembled that of the *rrs*-based phylogenetic tree (Fig. 3b and c). In the trees based on the *gltA* and *groEL* genes, all revealed sequences clustered together in the first lineage with some sequences of *A. capra* strains detected in goats, sheep, ticks, giraffes (previously defined as *A. phagoctophilum*), humans from China, and cattle from Japan (previously defined as *A. centrale*). For both *groEL* and *gltA* genes, the nucleotide identities in the first lineage were 99.5–100% between the seven sequences in this study and 99.3–99.9% between the known sequences of *A. capra* (Additional file 2: Table S1).

## Discussion

In the present study, we aimed to detect the *Anaplasma* species pathogenic to humans in ticks in Ankang city, Northwest China. Hard ticks, particularly *Ixodes persulcatus* and *Haemaphysalis longicornis*, are the main vectors that transmit *Anaplasma* species to humans and vertebrate animals by sucking blood [3, 9]. The primary vector for *A. platys* is considered to be *R. sanguineus* sensu lato [46–48]. The DNA of *A. capra* has been detected in *R. microplus* in Wuhan, China [36], whereas *A. platys-*like strains were also identified in *R. microplus* and *R. turanicus* [36, 49]. Here, *R. microplus* ticks were found infected with *A. capra* and *A. platys-*like strains by conventional PCR and sequencing. Co-infection by *A. platys*-like and *A. capra* was also observed. Hence, *R. microplus* may also be a vector for *A. capra* and *A. platys*-like strains, although further evidence is needed. Alternatively, these two pathogens may also be from animal hosts by sucking. Hence, their risk for humans is unknown and should be assessed in future studies.

*A. capra* was first detected in *I. persulcatus* in Heilongjiang Province, China, at 3% positive rate [3]. Subsequently, Sun et al. [9] identified it in *H. longicornis* in Shandong Province, China, at only 0.43% positive rate. Yang et al. [26] also found that *A. capra*-like strains circulated in *H. qinghaiensis* in Gansu Province, China, at 5.8% positive rate. Only 0.28% positive rate for *A. capra* was identified in *R. microplu*s in Wuhan [36]. In our previous study, the positive rate was 40.4% for *R. microplu*s and 22.6% for *H. longicornis* in Xi’an, China [29]. In this study, 36.6% (167/456) positive rate for *A. capra* was found in *R. microplus* ticks, similar to that for *R. microplu*s and *H. longicornis* in Xi’an but higher than that for *H. longicornis*, *H. qinghaiensis*, *I. persulcatus,* and *R. microplu*s in other previous studies [9, 26, 36]. *A. platys*-like strains were mainly detected in vertebrates rather than ticks. Only 1 of 354 *R. microplu*s ticks in Wuhan was positive for *A. platys-*like strains [36], which is less than 7.68% found for *R. microplu*s in this study.

Genetic and phylogenetic analysis based on the *rrs*, *gltA,* and *groEL* genes showed that specific *A. platys* strains formed a monophyletic group and shared more than 99.0% nucleotide identities regardless of whether they were collected from ticks, dogs, or other vertebrate animals, including humans. In recent years, several *A. platys*-like organisms were identified in cattle, sheep, goats, cats, and mosquitoes, sharing 86–93% similarity for the *groEL* gene with specific *A. platys* strains [35, 42, 43]. Furthermore, the *gltA* gene of *A. platys*-like strains from ticks and mosquitoes in China [35–37] formed a distinct lineage instead of clustering with specific *A. platys* strains (Fig. 2b). Another *A. platys*-like organism, detected in camels and named *Ca*. A. camelii [34, 50], clustered with specific *A. platys* strains in the *rrs* and *groEL* genes trees. In the *groEL* tree, *A. platys*-related strains detected in ticks from Ankang city had the closest relationship with strains recovered from mosquitoes [35], while only Ak-Rm383 collected in this study formed a distinct lineage in the *gltA* tree, clustering together with strains recovered from mosquitoes and ticks [35–37].

In this study, we used universal primers to obtain the *groEL* gene, and no sequencing results were ambiguous, which suggests that our tick samples were not co-infected with multiple strains of *A. platys* and related strains. Although we used primers specific to the *gltA* gene of the strains found in mosquitoes, we obtained only one sequence (data not shown), consistent with the results using the universal primers. Since the classification criteria for species of *Anaplasma* genus are not available, it is uncertain whether the lineages in the *gltA* and *groEL* phylogenetic trees, including *Ca*. A. camel, represent a distinct species or the same species.

In 2015, *A. capra* was considered as a new species and proved to be a human pathogen [3], although it has already been detected before in ticks and animals in China and Japan [37, 51–53]. After *A. capra* was considered to be pathogenic to humans, many molecular epidemiological investigations revealed its wide distribution in rural areas in China [9, 26, 35, 54]. The present study demonstrated the genetic diversity of *A. capra*. The first of two *A. capra* phylogenetic lineages was associated with the high positive rate in ticks. The strains described herein were most closely related to those detected in goats, sheep, various tick species, and humans based on the *rrs*, *gltA*, and *groEL* genes, revealing their genetic similarity. Hence, more attention should be paid to the infectivity of *A. capra* to humans.

The severity of the diseases caused by *Anaplasma* varies widely from mild (influenza-like illness) to severe (with lymphadenopathy and high hepatic aminotransferase concentrations). However, most cases caused by *A. capra* [3] and *A. platys* [5] mainly present as fever, headache, malaise, dizziness, and chills, and these nonspecific symptoms can easily be misdiagnosed as other infections [55]. A timely diagnosis of cases caused by *A. capra* is challenging for inexperienced clinicians, and laboratory identification is necessary under such circumstances. It is therefore urgent to develop rapid diagnostic procedures. Also, the pathogenicity of *A. platys*-like strains to humans should be determined in further studies.

## Conclusions

A survey of *Anaplasma* species pathogenic to human was conducted in ticks collected from domestic animals in Ankang, China. Our results showed that *A. capra* and *A. platys-*like strains are present in *R. microplus* in the local area although their transmission to humans is uncertain. *A. capra* appears to be especially prevalent in ticks, and the strains identified in the present study are genetically similar to those previously detected in humans. Consequently, it should receive more attention from researchers and clinicians working at disease prevention and control. The pathogenicity of *A. platys*-like strains characterized in this study should also be assessed further.

## Additional files

Additional file 1: **Figure S1.** The partial *gltA* and *groEL* genes of *A. platys*-like strains and different fragments of partial and nearly complete *rrs*, *gltA*, and *groEL* genes of *A. platys*-like strains and *A. capra* amplified with different pairs of primers from the tick DNA specimens. (TIF 360 kb)

Additional file 2: **Table S1.** Information for the sequences submitted to GenBank database used for phylogenetic analysis. (DOCX 18 kb)

## Abbreviations

HGA: Human granulocytic anaplasmosis
PCR: polymerase chain reaction
