# Supplementary figures and images for: Molecular identification and characterization of Anaplasma capra and Anaplasma platys-like in Rhipicephalus microplus in Ankang, Northwest China
Source: BMC Infect Dis. 2019 May 17;19:434. doi: 10.1186/s12879-019-4075-3 (PMC6525361; doi:10.1186/s12879-019-4075-3)

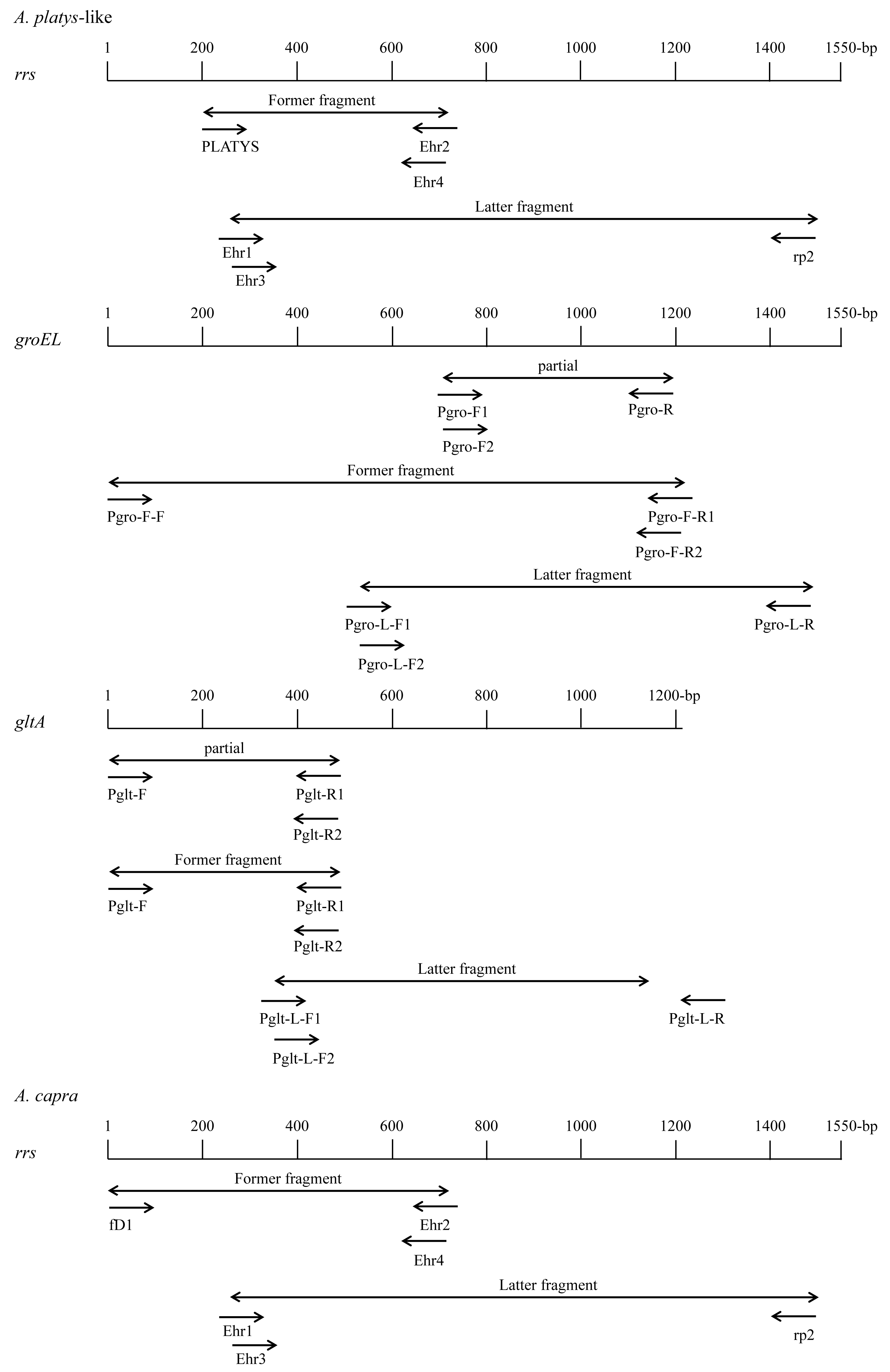

Supplement: Supplementary file 1 — Figure S1. The partial gltA and groEL genes of A. platys-like strains and different fragments of partial and nearly complete rrs, gltA, and groEL genes of A. platys-like strains and A. capra amplified with different pairs of primers from the tick DNA specimens. (TIF 360 kb) [file 12879_2019_4075_MOESM1_ESM.tif]
