# Supplementary material for: Molecular identification and characterization of Anaplasma capra and Anaplasma platys-like in Rhipicephalus microplus in Ankang, Northwest China
Source: BMC Infect Dis. 2019 May 17;19:434. doi: 10.1186/s12879-019-4075-3 (PMC6525361; doi:10.1186/s12879-019-4075-3)
Supplement: Supplementary file 2 — Table S1. Information for the sequences submitted to GenBank database used for phylogenetic analysis. (DOCX 18 kb) [file 12879_2019_4075_MOESM2_ESM.docx]

**Supplementary Table 1.** Information for the sequences submitted to GenBank database used for phylogenetic analysis

| Strains | *rrs* | | *gltA* | | *groEL* | |
| --- | --- | --- | --- | --- | --- | --- |
|  | Accession No. | Most  closely related sequences (% identity) | Accession No. | Most  closely related sequences (% identity) | Accession No. | Most  closely related sequences (% identity) |
| AK-Rm-3 | MH762078  (partial) | TTBR-HS, 100% | MH716428  (partial) | WSti2f, 75.3% | MH716436  (partial) | WHAEAL-17-2, 100% |
| AK-Rm-113 | MH762084  (partial) | D35, 99.9% | MH716424  (partial) | WSti2f, 75.6% | MH716432  (partial) | WHAEAL-17-2, 100% |
| AK-Rm-228 | MH762079 | D35, 99.9% | MH716426 | WSti2f, 77.8% | MH716434 | WHANSL-27-1, 99.0% |
| AK-Rm-259 | MH762080 | D35, 99.9% | MH716427  (partial) | WSti2f, 75.6% | MH716435 | WHANSL-27-1, 98.7% |
| AK-Rm-341 | MH762083  (partial) | D35, 99.9% | MH716421  (partial) | WSti2f, 75.5% | MH716429 | WHANSL-27-1, 98.8% |
| AK-Rm-383 | MH762085  (partial) | D35, 100% | MH716425  (partial) | Uncultured Anaplasma sp. clone SY124, 97.9% | MH716433  (partial) | WHAEAL-17-2, 100% |
| AK-Rm-403 | MH762081 | D35, 99.9% | MH716422 | WSti2f, 77.4% | MH716430 | WHANSL-27-1, 99.0% |
| AK-Rm-420 | MH762082 | D35, 99.9% | MH716423  (partial) | WSti2f, 75.7% | MH716431 | WHANSL-27-1, 98.9% |
| AK-Rm-187 | MH762071 | Anaplasma sp. A60, 100% | MH716407 | Anaplasma sp. strain WHBMXZ-125, 99.8% | MH716414 | Anaplasma sp. strain WHBMXZ-125, 99.9% |
| AK-Rm-235 | MH762072 | Anaplasma sp. A60, 99.9% | MH716408 | Anaplasma sp. strain WHBMXZ-125, 99.8% | MH716415 | Anaplasma sp. strain WHBMXZ-125, 99.9% |

*(continued)*

| Strains | *rrs* | | *gltA* | | *groEL* | |
| --- | --- | --- | --- | --- | --- | --- |
|  | Accession No. | Most  closely related sequences (% identity) | Accession No. | Most  closely related sequences (% identity) | Accession No. | Most  closely related sequences (% identity) |
| AK-Rm-277 | MH762073 | Anaplasma sp. A60, 99.8% | MH716409 | Anaplasma sp. strain WHBMXZ-125, 99.8% | MH716416 | Anaplasma sp. strain WHBMXZ-125, 99.6% |
| AK-Rm-309 | MH762074 | Anaplasma sp. A60, 99.9% | MH716410 | Anaplasma sp. strain WHBMXZ-125, 99.8% | MH716417 | Anaplasma sp. strain WHBMXZ-125, 99.9% |
| AK-Rm-337 | MH762075 | Anaplasma sp. A60, 99.9% | MH716411 | Anaplasma sp. strain WHBMXZ-125, 99.8% | MH716418 | Anaplasma sp. strain WHBMXZ-125, 99.9% |
| AK-Rm-380 | MH762076 | Anaplasma sp. A60, 99.9% | MH716412 | Anaplasma sp. strain WHBMXZ-125, 99.8% | MH716419 | Anaplasma sp. strain WHBMXZ-125, 99.9% |
| AK-Rm-429 | MH762077 | Anaplasma sp. A60, 100% | MH716413 | tick-XA143, 100% | MH716420 | Anaplasma sp. strain WHBMXZ-125, 99.9% |
